# A mycobacterial Sec61 inhibitor disrupts lysosome function by blocking Vacuolar-ATPase biosynthesis

**DOI:** 10.1016/j.ejcb.2026.151541

**Published:** 2026-09

**Authors:** Belinda S. Hall, Kwabena Owusu-Boateng, Kerry L. McPhail, Wei Q. Shi, Rachel E. Simmonds

**Affiliations:** a Discipline of Microbes, Infection and Immunity, School of Bioscience and Medicine, Faculty of Health and Medical Science, University of Surrey, Guildford, UK; b Department of Medicinal Chemistry, L. S. Skaggs Pharmacy Institute, Salt Lake City, UT, USA; c Department of Chemistry, Ball State University, Muncie, IN, USA

**Keywords:** Lysosome/Vacuolar-ATPase/mycolactone/Sec61

## Abstract

Mycolactone is the virulence toxin of *Mycobacterium ulcerans*, causative agent of Buruli ulcer. Mycolactone inhibits the Sec61-dependent co-translational translocation of signal peptide-bearing secreted and membrane proteins into the endoplasmic reticulum. Sec61 inhibition leads to accumulation of mislocalised proteins in the cytosol and initially triggers an integrated stress response-dependent activation of autophagy that contributes to cell survival. Here we show sustained exposure to mycolactone blocks late-stage autophagy and induces nuclear translocation of the lysosomal stress marker TFEB. This follows loss of ATP6AP1 and ATP6AP2, Sec61-substrates required for assembly of the Vacuolar-ATPase, leading to reduced lysosomal biogenesis and acidification. These effects are reduced in cells expressing a mycolactone-resistant Sec61α mutant and phenocopied by other Sec61 inhibitors. Loss of lysosomal function compromises the cell’s capacity to withstand the proteostatic stress caused by Sec61 inhibition and could impair the ability of phagocytes to combat infection with *M. ulcerans* and contribute to the tissue necrosis in Buruli ulcer. Furthermore, since Sec61 inhibition is being pursued as a therapeutic target in several diseases, potential drugs should be screened against this activity to avoid unwanted side-effects.

## Highlights

• Long term Sec61 inhibition by *M. ulcerans* toxin mycolactone reduces lysosomal activity and blocks late-stage autophagy.
• Mycolactone rapidly downregulates the V-ATPase accessory proteins ATP6AP1 and ATP6AP2.
• Loss of ATP6AP1/2 leads to disrupted V_1_ complex association with lysosomes and reduced production of acidic lysosomes.
• This compromises proteostatic stress responses and could contribute to the cytotoxicity seen in Buruli ulcer lesions.

## Graphical Abstract

**Figure:**
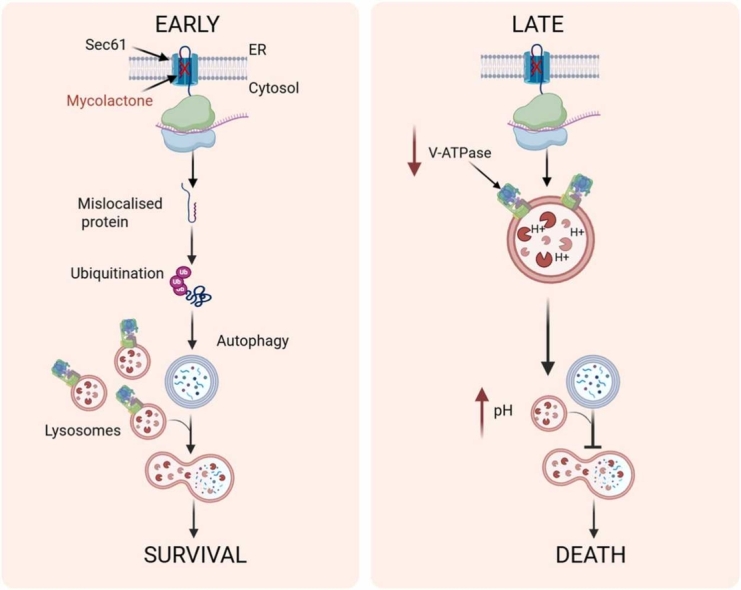

## 1 Introduction

Buruli ulcer is a chronic skin infection caused by *Mycobacterium ulcerans* characterised by extensive tissue necrosis with little inflammation. Mycolactone, a polyketide lactone with cytotoxic, immunosuppressive and analgesic properties, is the sole virulence factor of *M. ulcerans* and is responsible for all disease pathology (Baron et al., 2016, George et al., 1999, George et al., 2000, Guenin-Mace et al., 2011, Marion et al., 2014, Yotsu et al., 2018). The main target of mycolactone is the Sec61 translocon, an oligomeric complex which translocates nascent polypeptides co-translationally into the endoplasmic reticulum (ER) (Bhadra et al., 2021, Hall et al., 2014). Mycolactone binds directly to Sec61α, blocking access of signal-peptide bearing proteins to the channel, and preventing opening of its plug domain (Gerard et al., 2020, Itskanov et al., 2023). As a result, translocation of almost all secreted proteins, Type I and II single-pass transmembrane proteins, and a small subset of multi-pass membrane proteins is inhibited (Hsieh et al., 2025, McKenna et al., 2017).

Sec61 inhibition by mycolactone explains the immunosuppression seen in Buruli ulcer because immune cells cannot produce the receptors and cytokines needed for innate and acquired immune responses. It also drives mycolactone’s cytotoxicity, since cells expressing non-mycolactone binding Sec61α mutants display resistance. However, the cellular mechanisms causing cell death consequential to Sec61 inhibition remain poorly defined.

Mycolactone-sensitive membrane proteins are translated at the inhibited ribosome-translocon complex but are made in the cytosol. These mislocalised proteins are a potential source of proteotoxic stress and are ubiquitinated then degraded by the proteasome or selective autophagy, with the latter regulated by the integrated stress response (ISR) rather than the classic ULK-dependent pathway (Hall et al., 2021). Inhibition of autophagy or the ISR leads to rapid cell death (Ogbechi et al., 2018), suggesting this is a protective response and upregulation of autophagic flux promotes cell survival.

However, a complex cellular phenotype evolves over time. Following initial exposure to mycolactone, puncta positive for early autophagic markers WIPI2, ATG16L1 and FIP200 increase, and increased flux is detected in assays using the tandem tag probe (mCherry-eGFP-LC3) (Hall et al., 2021, Pankiv et al., 2007) but, after 24hrs, evidence of autophagy inhibition emerges, with accumulation of the adaptor protein SQSTM1/p62 suggesting late stage autophagy is blocked (Klionsky et al., 2021). Despite increased SQSTM1/p62 production via transcriptional and translational upregulation, this appears to represent a different phase of the response (Hall et al., 2021). We therefore postulated that mycolactone may impact lysosomal function over the longer term, counteracting the protective role of autophagy and driving eventual cell death.

## 2 Materials and methods

### 2.1 Mycolactone

For all experiments, we used synthetic mycolactone A/B, which was generously donated by Prof. Yoshito Kishi (Harvard University). To control for potential impact of the DMSO solvent on cell function, DMSO diluted equivalently was used; typically this was 0.02%.

### 2.2 Cell culture and treatment

HeLa and L929 cells were maintained in DMEM supplemented with 10% FBS at 37°C and 5% CO_2_. HeLa cells constitutively expressing Sec61α D60G (Ogbechi et al., 2018) were maintained in the presence of 400 μg/ml Hygromycin B (Fisher Scientific). To generate conditioned medium, L929 cells were cultured to confluency, then maintained for 3 further days. Supernatants were collected and spun at 300 x g for 5 min, then filtered and stored at −70⁰C. Juvenile, single donor human microvascular endothelial cells (HDMEC) were cultured in hVEGF containing Endothelial cell growth medium 2 (Promocell) at 37°C and 5% CO_2_. Cells were routinely seeded at a concentration of 1 × 10^4^/cm^2^ in 25 cm^2^ or 75 cm^2^ flasks for no more than 15 population doublings. THP-1 cells were cultured in suspension in RPMI supplemented with 10% FBS at 37°C and 5% CO_2_. Cell density was maintained between 0.1 and 10^5^ cells/ml. For differentiation, cells were plated out at a density of 5 × 10^5^/ml in the presence of 20 ng/ml phorbol myristate acetate (PMA) for 72 hr. Medium was replaced and the cells allowed to rest for 24 hr before experiments. Bone marrow derived macrophages (BMDM) derived from C57BL/6 J mice were differentiated for 7 days in 80% RPMI supplemented with 10% FBS/20% L929 conditioned medium. After harvesting cells were plated out at a density of 5 × 10^5^/ml. Mycolactone was used at a concentration of 31.25 ng/ml for HeLa cells, THP-1 and BMDM and at 10 ng/ml for HDMEC. Bafilomycin A1 (BAFA1) was used at 300 nM for HeLa cells and 200 nM for all other lines. ZIF-80 (Zong et al., 2020) and Coibamide A (CbA) (Tranter et al., 2020) were used at 100 nM and 250 nM, respectively, for 24 hr. The mTOR inhibitor PP242 (Merck) was used at 10 μM for 1 hr.

### 2.3 Transfections

The tandem tag vector pDEST-mCherry-eGFP-LC3B was a gift from Terje Johansen and pYMA1_SidK_mCH was a gift from John Rubinstein (Addgene plasmid #184906; http://n2t.net/addgene:184906; RRID:Addgene_184906). Cells in 80% confluent 6-well plates were transfected overnight with 2 µg plasmid DNA then trypsinised and plated onto coverslips for co-staining with antibodies or IBIDI chamber slides for live cell imaging. Cells were incubated overnight then with DMSO, mycolactone or BAFA1 as described then pDEST-mCherry-eGFP-LC3B transfected cells were imaged live while pYMA1_SidK_mCH transfected cells were fixed for immunofluorescent staining.

### 2.4 Immunoblotting

Immunoblotting was carried out according to standard methods as described (Ogbechi et al., 2018). Cells were plated onto 6 well plates, incubated with mycolactone for various times then lysed directly in 1 x Laemmli SDS sample buffer (with sonication to degrade genomic DNA). Proteins were separated on 4–20% pre-cast gels (BioRad) and blotted onto Immobilon PVDF membranes (Merck). Blots were blocked with 5% milk powder in TBS/0.05% Tween 20. Primary antibodies were diluted in 3% BSA/TBS/0.05% Tween 20). Antibodies used in this study were: Anti-ATP6AP1 (Santa Cruz Biotechnology, sc-81886); anti-ATP6AP2 (Bio-Techne, NBP1–90820); anti-ATP6V0D1 (Proteintech, Proteintech: 18274–1-AP); anti-ATP6V1H (Cambridge Bioscience, A304–308A),anti-GAPDH (Fisher Scientific, 10515025), anti-LAMP1 (Cell Signaling Technologies, 15665), anti-Rab7 (Merck, R8779), anti-phospho-TFEB (Ser211) (Cell Signaling Technologies, 37681) and anti-TFEB (Proteintech 68632). Secondary antibodies were anti-rabbit-HRP (GE Healthcare, NA934V) and anti-mouse-HRP (GE Healthcare, NA931V) and protein bands were visualised using Immobilon Western Chemiluminscence Substrate (Fisher Scientific). Images were developed on a Fusion FX Imager (Vilber-Lourmatclean), which provides a warning if the areas of the image are saturated.

### 2.5 Immunofluorescence

Immunofluorescent imaging was carried out according to standard methods as described (Hall et al., 2021). Cells were plated onto sterile glass coverslips and incubated overnight before experiments. Cells were either fixed with ice-cold methanol and blocked with 2% BSA/PBS overnight before staining or fixed with 4% paraformaldehyde in 200 mM Hepes pH7.5 then permeabilised with 0.25% Nonidet P-40 alternative in NETGEL buffer (150 mM NaCl, 5 mM EDTA, 50 mM Tris-Cl, pH 7.4, 0.05% Nonidet P-40 alternative, 0.25% gelatin and 0.02% sodium azide) and blocked with NETGEL buffer. Antibodies used in this study were: anti-TFEB (Cambridge Bioscience, A303–673A); anti-human LAMP1 (Bio-Techne, MAB4800): anti-ATP6V1D (Abcam, ab157458) and anti-Giantin (Abcam, ab80864). Secondary antibodies were: Alexa Fluor 594 goat anti-rabbit (A11012); Alexa Fluor 488 donkey anti-mouse (A21202); Alexa Fluor 488 goat anti-rabbit (A11034); Alexa Fluor 647 goat anti-rabbit (A21244) and Alexa Fluor 647 goat anti-mouse 647 (A21235), all from Fisher Scientific. Slides were counterstained with 0.1 μg/ml DAPI and mounted with Polymount (Tebu-Bio).

### 2.6 DQ Red BSA assay

Cells were plated onto IBIDI 8-well chamber slides and incubated overnight then incubated with 0.05% DMSO for 32 hr, 300 nM BAFA1 for 4 hr or 31.25 ng/ml mycolactone for various times. DQ Red BSA (20 μg/ml) (Fisher Scientific) was added in Optimem (Fisher Scientific) 4 hr before the end of the experiment. Cells were gently washed three times with Live Cell Imaging Medium (Fisher Scientific) and returned to the incubator for 5 min before imaging.

### 2.7 Alexa Fluor 647 Dextran labelling

Cells plated onto coverslips were incubated overnight then with 0.05% DMSO for 32 hr, 300 nM BAFA1 for 4 hr or 31.25 ng/ml mycolactone for various times. Alexa Fluor 647 Dextran 10,000 (0.1 mg/ml) was added for three hours, then cells were washed and incubated for 1 hr in fresh medium. Cells were fixed and mounted on Polymount for imaging

### 2.8 Lysotracker labelling

Cells were plated onto IBIDI 8-well chamber slides and incubated overnight then incubated with 0.05% DMSO for 32 hr, 300 nM BAFA1 for 4 hr or 31.25 ng/ml mycolactone for various times then incubated for 10 min with Lysotracker Green DND-26 (Fisher Scientific). Cells were gently washed three times with Live Cell Imaging Medium (Fisher Scientific) and returned to the incubator for 10 min before imaging live.

### 2.9 Acridine orange

Cells were plated onto IBIDI 8-well chamber slides and incubated overnight then incubated with 0.05% DMSO for 32 hr, 300 nM BAFA1 for 4 hr or 31.25 ng/ml mycolactone for various times. Acridine orange (2 μM, Bio-Rad) was added for 30 min, cells were washed three times with Live Cell Imaging Medium (Fisher Scientific) and imaged live.

### 2.10 Imaging and analysis

All imaging was carried out on a Nikon Eclipse TI confocal microscope. All image analysis was carried out using ImageJ software. Puncta were counted manually for the tandem tag analysis and using the Analyse Particles function for all other assays. The Measure function was used to quantify mean and integrated density fluorescence per cell. Co-localisation analysis was carried out using the ImageJ Coloc 2 function.

### 2.11 Statistical analysis

All data were analysed using GraphPad Prism Version 10.4.1. Data were analysed using a one-sample *t*-test, or one- or two-way ANOVA using pairing if appropriate. Where data were not normally distributed, a non-parametric test (Kruskal-Wallis) was used. An appropriate correction for multiple comparisons (either Dunnett’s, Tukey’s or Dunn’s) was performed if possible.

## 3 Results and Discussion

### 3.1 Mycolactone disrupts lysosomal function

**Fig. 1:**
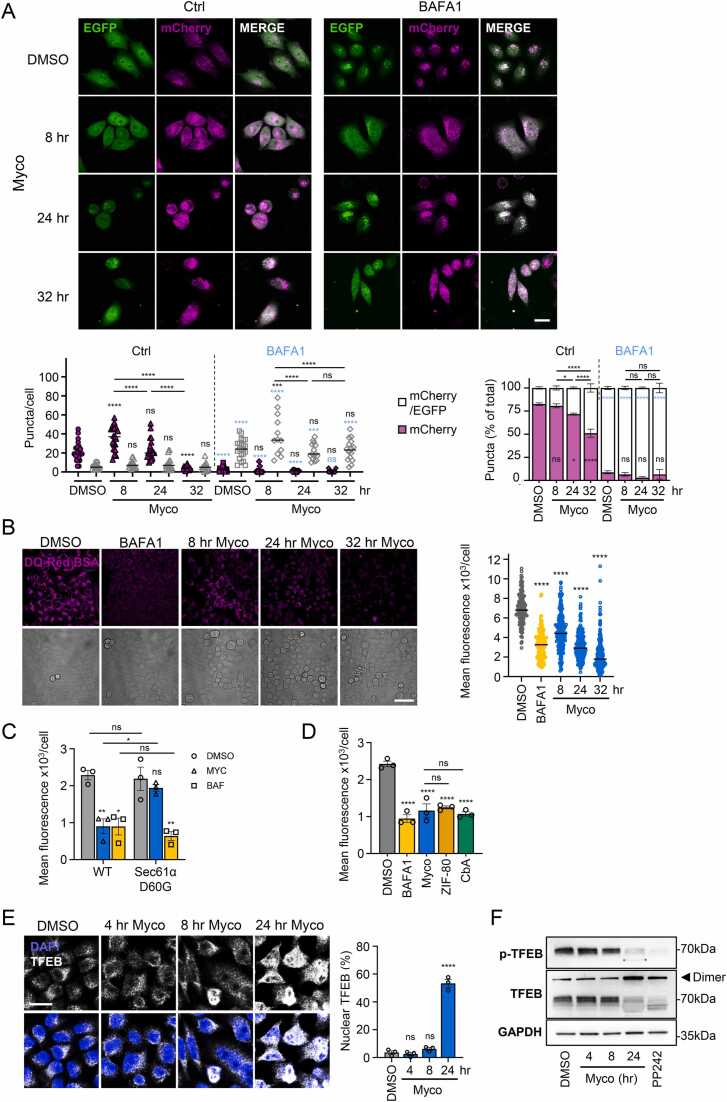
Mycolactone blocks late-stage autophagy, disrupts lysosomal function and activates TFEB signalling pathway. **A-E.** HeLa cells were incubated with 0.05% DMSO for 32 hr, 31.25 ng/ml mycolactone (Myco), 100 nM Zif80 or 250 nM Coibaminde A (CbA) for various times as indicated with or without 300 nM Bafilomycin A1 (BAFA1) for 4 hr. **A.** Cells transiently transfected with pDEST-mcherry-eGFP-LC3B before treatment with mycolactone as described followed by live cell imaging, with the mCherry fluorescence pseudocoloured as magenta and eGFP as green. Quantitation shows the total number per cell (left hand graph, data representative of n = 3 independent experiments; ordinary 2-way ANOVA) and relative proportions of magenta and white puncta (right hand graph, mean of n = 3 biological replicates ± SEM; RM 2-way ANOVA) visualised in cells expressing mCherry-eGFP-LC3B. Scale bar = 20 µm. Data are compared to DMSO in the absence and presence of BAFA1 separately, and the effect of BAFA1 on each treatment is compared (indicated in blue). **B.** Cells were incubated with 20 µg/ml DQ Red BSA for 4 hr before washing and imaging for the unquenched signal. Scale bar = 50 µm. Quantitation of mean fluorescence per cell for at least 178 cells (data representative of n = 3 independent experiments, ordinary 1-way ANOVA).**C.** WT or Sec61α D60G expressing Hela cells were incubated for 20 hr with DMSO, Myco or BAFA1 then with 20 µg/ml DQ Red BSA for 4 hr before washing and imaging. Fluorescence was quantified for at least 224 cells over 3 fields. Data represents the mean fluorescence values for n = 3 independent experiments ± SEM (ordinary 2-way ANOVA) **D.** Hela cells were incubated for 20 hr with DMSO, Myco, ZIF-80, CbA or BAFA1 then with 20 µg/ml DQ Red BSA for 4 hr before washing and imaging. Fluorescence was quantified for at least 249 cells over 3 fields. Data represents the mean fluorescence values for n = 3 independent experiments ± SEM. **E.** Cells were fixed with 4% PFA, permeabilised, stained with anti-TFEB antibodies (grey) and counterstained with DAPI. Scale bar = 20 µm. Graph shows mean % cells with nuclear TFEB staining from n = 3 biological replicates ± SEM (1-way ANOVA). **F.** Immunoblots from Hela cells incubated with DMSO, PP242 or mycolactone for various times as and stained with the antibodies indicated. GAPDH is included as a loading control. Data is representative of n = 3 independent experiments. ns, not significant; *,p < 0.05; **, p < 0.01; ***, p < 0.001; ****, p < 0.0001.

To assess the longer-term impact of mycolactone on autophagic flux, we used the same tandem-tag probe as previous investigations (Hall et al., 2021) (mCherry-eGFP-LC3, FigS1A), over an extended range of timepoints (Fig1A). In cells with a functioning autophagy pathway, LC3-labelled autophagosomes form only transiently before fusing with the lysosome. Since the low lysosomal pH quenches the eGFP signal, these autolysosomes are identified as mCherry^+ve^ puncta (magenta in Fig1A), while unfused autophagosomes are mCherry^+ve^/eGFP^+ve^ (white in Fig1A). Changes to flux are revealed when cells are exposed to the Vacuolar-ATPase (V-ATPase) inhibitor BAFA1, which prevents lysosomal acidification, leading to an increase in dual-coloured puncta that is enhanced by increased flux (Bowman et al., 1988, Klionsky et al., 2021, Pankiv et al., 2007).

Exposure to mycolactone for 8 hrs caused significantly increased numbers of mCherry^+ve^/eGFP^+ve^ (white) autophagosomes in the presence of BAFA1, confirming an early increase in autophagy flux (Fig1A, Fig S1B). However, at later timepoints (24 and 32 hrs) this effect was reversed. Notably, at later timepoints the number of mCherry^+ve^ lysosomes (magenta) also decreased, and by 32hrs was extremely low. At this stage, the relative abundance of mCherry^+ve^ and mCherry^+ve^/eGFP^+ve^ puncta expressed as a proportion (magenta:white) had also decreased significantly, with approximately 50% of puncta dual-labelled, suggesting inhibition of fusion with lysosomes and/or increased lysosomal pH (Fig. 1A, lower panels). However, since BAFA1 could still increase the number of mCherry^+ve^/eGFP^+ve^ (white) autophagosomes at 32 hr, the effect was only partial. This shows that after an initial increase in autophagic flux, prolonged exposure to mycolactone interferes with late-stage autophagy. This resolves the apparent discrepancy between our previous finding that mycolactone activates autophagy and an earlier report that it inhibits the pathway (Gama et al., 2014).

This effect was not due to cell death as cells remained ∼90% viable for up to 48 hr in the presence of mycolactone, and significant levels of apoptotic death were only seen after 72 hr (Fig S1C). Notably, a structurally distinct Sec61 inhibitor, CbA, induces a similar response, causing initial upregulation of autophagy but inhibition at later stages, supporting translocon inhibition as the underlying cause (Hau et al., 2013, Shi et al., 2021, Tranter et al., 2020).

One explanation for these observations could be loss of lysosomal function, since this is required to complete the process of autophagy. To determine whether mycolactone alters lysosomal proteolytic digestion capacity, HeLa cells were incubated with DQ Red BSA, a fluorogenic protease substrate which is de-quenched following proteolysis, yielding a strong fluorescent signal in lysosomes. In control cells, high levels of fluorescence were detected in all cells (Fig. 1B) while the signal was blocked by BAFA1 due to loss of lysosome acidification. Mycolactone caused a progressive decrease in DQ red BSA fluorescence which was statistically significant as early as 8 hr after exposure and lower than that seen in BAFA1-treated cells by 32hrs. This is not specific to HeLa cells as similar results were obtained in primary murine BMDM and human macrophage-like cells (PMA-activated THP-1 cells) (Fig S1D, S1E).

Notably, mycolactone failed to block DQ Red BSA proteolysis in cells expressing the Sec61α mutant D60G which confers resistance to mycolactone (Ogbechi et al., 2018) (Figs1C, S1F) and similar results were obtained using the alternative Sec61 inhibitors ZIF-80 and CbA (Figs1D, S1G), demonstrating that Sec61 inhibition causes a general suppression of lysosomal function and confirming that these effects are due to inhibition of Sec61-dependent translocation.

The transcription factor TFEB controls autophagy and lysosomal biogenesis by upregulating genes in the Coordinated Lysosomal Expression and Regulation (CLEAR) network (Sardiello et al., 2009). TFEB activation and nuclear translocation follows loss of mTORC-dependent phosphorylation triggered by multiple pathways such as nutrient deprivation and metabolic or oxidative stress, or following lysosomal dysfunction caused by lysosomal storage disorders, membrane damage, calcium loss or changes in luminal pH (Pena-Llopis et al., 2011, Raben and Puertollano, 2016). In control cells, TFEB was predominantly cytoplasmic and little change could be seen up to 8 hr after mycolactone addition but by 24 hr over 50% of nuclei were positive for TFEB (Fig. 1E). Loss of TFEB phosphorylation at the TORC1 site Ser211 was confirmed by immunoblotting, which revealed a reduction in phosho-TFEB at 24 hr paralleled by an increase in the unphosphorylated active dimer form, although the effect was not as complete as that achieved with the mTOR inhibitor PP242 (Fig. 1F). This is in line with our earlier findings that mTOR inactivation (evidenced by changes in ULK1 and AKT phosphorylation) is a relatively late-stage event following exposure to mycolactone (Hall et al., 2021, Ogbechi et al., 2018). Nuclear translocation of TFEB was significantly reduced in cells expressing Sec61α D60G (Fig S1H-I) while exposure to ZIF-80 or CbA (Fig S1J-K) caused an increase comparable to that seen with mycolactone. Taken together the data suggest long term Sec61 inhibition triggers lysosomal stress.

### 3.2 Mycolactone rapidly downregulates the V-ATPase accessory subunits ATP6AP1 and ATP6AP2

Lysosome function depends on luminal and membrane proteins that are dependent on Sec61 for translocation into the ER. Such constitutively expressed proteins are depleted at their turnover rate following Sec61 inhibition (Ogbechi et al., 2015) but the rate of depletion of individual proteins is variable. In our recent proteomic study of HDMEC (Hsieh et al., 2025), we detected 138 lysosome-associated proteins, of which 24 showed reduced expression after 24 hr mycolactone exposure (PRIDE Project PXD037489). However, only two proteases, legumain and Cathepsin S, were significantly downregulated at this point. Although we cannot rule out synergistic effects of the combined loss of multiple proteins, loss of proteases alone seems to be insufficient to explain the impact of mycolactone on lysosome function. We also detected no change in levels of Rab7, downregulation of which would be expected to reduce endosome-lysosome fusion and impair autophagy (Fig S2A).

**Fig. 2:**
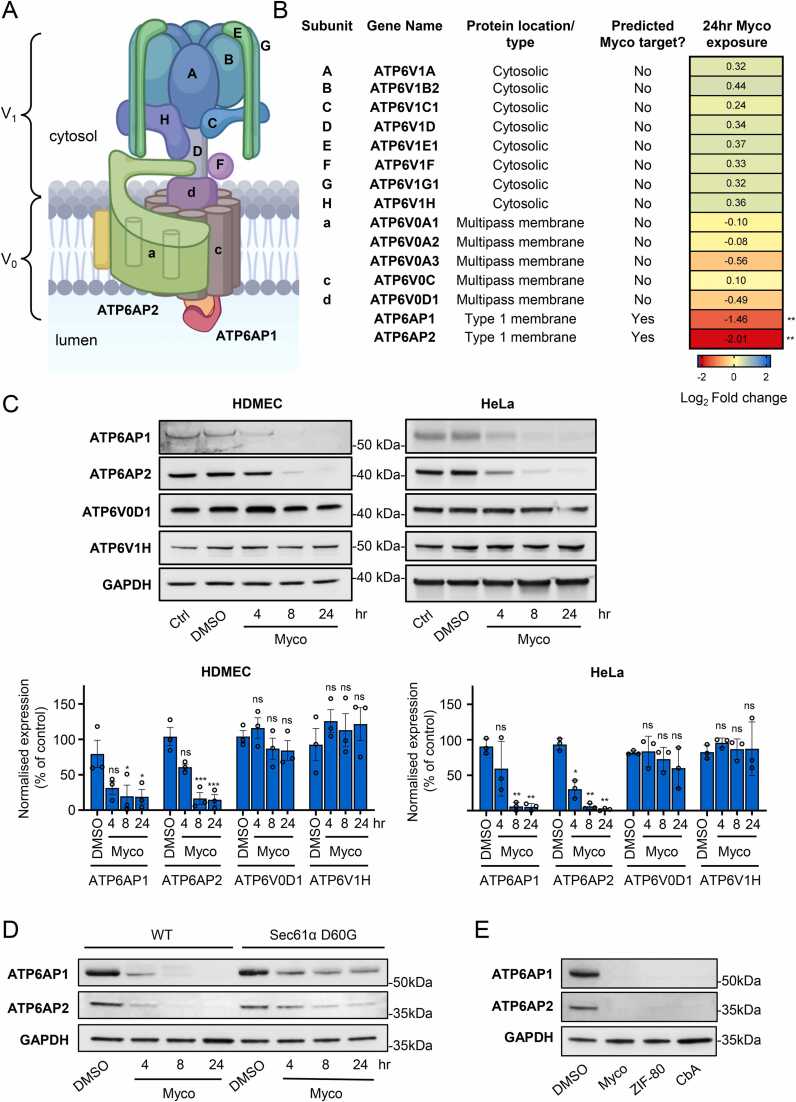
Mycolactone targets the ATP6A1 and ATP6A2 subunits of the Vacuolar ATPase. **A.** Cartoon structure of V-ATPase complex, depicting the cytosolic V_1_ complex responsible for ATP hydrolysis and the membrane embedded V_0_ which pumps protons across the membrane. Image generated using Biorender. **B.** V-ATPase subunits detected in our HDMEC membrane proteome (PRIDE Project PXD037489)(Hsieh et al., 2025) showing predicted sensitivity to mycolactone based on location and topology combined with the known specificity of mycolactone towards different Sec61 substrates, along with a heat map of Log2 fold-change in V-ATPase subunit expression following mycolactone exposure for 24 hr. **, p < 0.01. **C.** Immunoblots from HDMEC incubated with 0.02% DMSO or 10 ng/ml mycolactone and HeLa cells incubated with 0.05% DMSO for 24 hr or 31.25 ng/ml mycolactone for various times and stained with the indicated antibodies. Quantitation of band intensity relative to GAPDH is shown for each cell type. Data represents the mean of n = 3 independent experiments ± SEM (1-way ANOVA). ns, not significant; *, p < 0.05; **, p < 0.01; ***, p < 0.001. **D**. Immunoblots from WT and Sec61α D60G expressing HeLa cells incubated with with 0.05% DMSO for 24 hr or 31.25 ng/ml mycolactone for various times and stained with the indicated antibodies. **E**. Immunoblots from HeLa cells incubated with with 0.05% DMSO, 31.25 ng/ml mycolactone, 100 nM ZIF-80 or 250 nM CbA for 24 hr. For all blots GAPDH is included as a loading control. Approximate molecular weights in KDa are indicated for each target. Images are representative of n = 3 independent experiments.

We therefore turned our attention to the V-ATPase, which regulates lysosome acidity (as well as that of Golgi and endosomes) and thus controls the activity of many lysosomal enzymes. The V-ATPase is a complex made up of two multi-subunit domains, the cytosolic V_1_, responsible for ATP hydrolysis, and the membrane-integral V_0_, which translocates protons across the membrane (Fig. 2A) (Abbas et al., 2020, Nelson and Harvey, 1999, Wang et al., 2020). The proteins making up the V-ATPase complex, and their predicted and actual sensitivity to mycolactone are shown in Fig. 2B. The cytosolic V_1_ proteins and the multipass components of the V_0_ complex are not predicted targets of mycolactone due to their location and topology. Their abundance in the proteome was not significantly changed after 24 hr mycolactone exposure (Fig. 2B) which we confirmed by blotting for ATP6V0D1 and ATP6V1H in both HDMEC and HeLa cells (Fig. 2C). However, the two type I membrane proteins, ATP6AP1 and ATP6AP2, are predicted targets of mycolactone and were significantly depleted following 24 hr exposure (Fig. 2B). We explored the kinetics of this depletion and found both ATP6AP1 and ATP6AP2 are rapidly lost in the presence of mycolactone in primary HDMEC, HeLa (Fig2C) and PMA-differentiated THP-1 (Fig S2B) cells, with a reduction detectable as early as 4 hr post-exposure. Reduced levels of both ATP6AP1 and ATP6AP2 were detected in HeLa cells expressing Sec61α D60G after mycolactone exposure, but expression of both proteins remained significantly higher than in wild-type cells at all time points (Fig 2D and S2C). Cells exposed to either ZIF-80 or CbA for 24 hr showed similar levels of depletion of the V-ATPase accessory proteins to mycolactone treated cells (Fig 2E and S2D)

ATP6AP1 and ATP6AP2 are accessory proteins of the V-ATPase. While not directly involved in proton translocation, they are essential for biogenesis of functional V- ATPase complexes and are important for lysosome function and production (Abbas et al., 2020, Nelson and Harvey, 1999, Wang et al., 2020). The two appear to act in concert, with ATP6AP1 acting as a hub around which the V_0_ membrane components assemble in the ER, controlling both stability and trafficking of the complex (Abbas et al., 2020, Guida et al., 2018, Wang et al., 2020). Their knockdown, deletion or mutation has been shown to reduce V_0_ protein levels and increase lysosomal and endosomal pH (Pareja et al., 2018). Moreover, depletion of either ATP6AP1 or ATP6AP2 alone can reduce lysosome number, decrease cargo degradation and disrupt autophagy (Pareja et al., 2018). ATP6AP1 may even play a direct role in autophagosome-lysosome fusion via interaction with Rab7 and the HOPS complex (Yan et al., 2025). Genetic disorders caused by mutations in ATP6AP1 and ATP6AP2 have pleiotropic effects on multiple tissues while altered expression and loss of function mutations are seen in many cancers (Jansen et al., 2016, Pareja et al., 2018, Qi et al., 2022, Rujano et al., 2017). Their downregulation by mycolactone would be expected to affect multiple cellular functions but particularly impact lysosomes and lysosome dependent processes.

### 3.3 Mycolactone reduces association of V1 complex with lysosomes

**Fig. 3:**
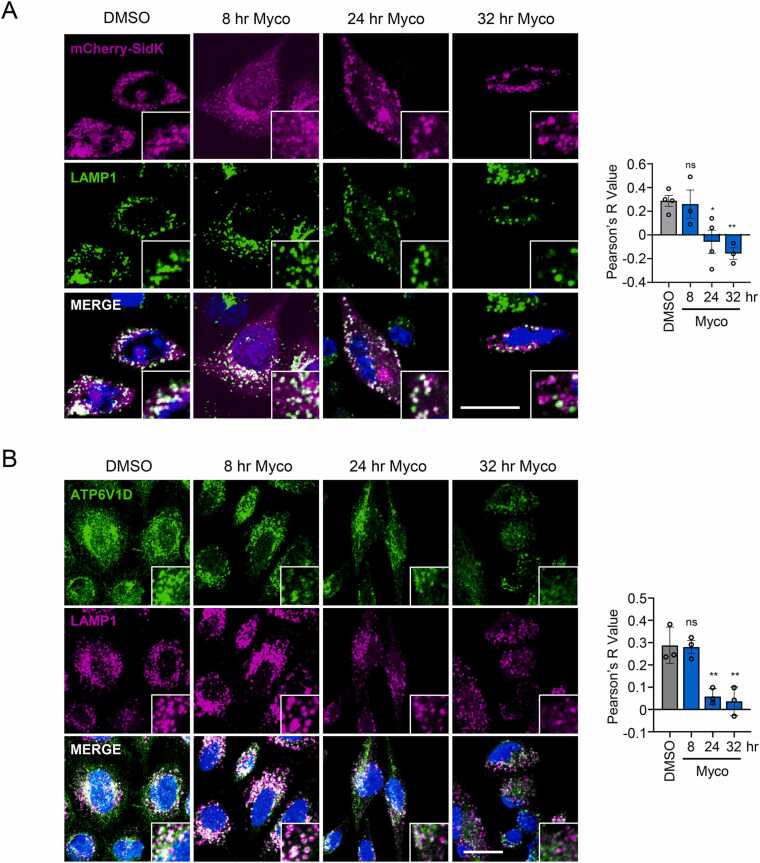
Reduced association of V-ATPase V_1_ with lysosomes. HeLa cells were incubated with 0.05% DMSO for 32 hr or 31.25 ng/ml mycolactone (Myco) for various times. All cells were counterstaining with DAPI; scale bar = 20 µm. Images representative of n = 3 independent experiments. **A.** Cells were transiently transfected with pYMA1_SidK_mCherry (pseudocoloured in magenta) prior to the experiment, and fixed/stained with anti-human LAMP1 antibodies (green). Quantitation shows mean Pearsons R value above threshold in transfected cells from at least n = 4 independent experiments±SEM (1-way ANOVA). **B.** Cells were fixed/stained with anti-ATP6V1D (green) and anti-human LAMP1 antibodies (magenta). Quantitation shows mean Pearsons R value above threshold from n = 3 independent experiments±SEM (1-way ANOVA). ns, not significant; *, p < 0.05; **, p < 0.01.

SidK is a bacterial toxin which binds specifically to the cytoplasmic ATP6V1A subunit of the V-ATPase (Xu et al., 2010) and expression of fluorescently-tagged SidK truncation mutants can be used to localise and quantify functional V-ATPase complexes in cells (Maxson et al., 2022). To investigate the effect of mycolactone on the V-ATPase complex, we used a SidK-mCherry construct (magenta in Fig. 3A) and examined co-localisation with LAMP1 (green in Fig3A). In control cells, some regions were positive for SidK-mCherry but not LAMP1, likely V-ATPase associated with Golgi and endosomal membranes. However, the majority of LAMP1^+ve^ compartments coincided with SidK-mCherry (white in Fig3A). The same pattern of staining was observed in cells exposed to mycolactone for at 8 hr. However, by 24 hr the association was reduced and after 32 hr mycolactone exposure, there was little overlap of signals, with cells exhibiting a redistribution of both LAMP1 and SidK-mCherry positivity from the perinuclear region to the cell periphery. These effects were confirmed by quantitative analysis of the degree of colocalization using Pearson’s R value. Since SidK associates with multiple compartments, the R value for DMSO treated cells is relatively low, however a clear drop into negative values, indicating reduced colocalization, is seen after 24 hr. The effect of mycolactone on SidK localisation was not just a consequence of altered LAMP1 expression as a similar effect was observed in cells labelled with Alexa 647 Dextran, which accumulates in lysosomes and provides and independent measure of lysosome numbers (Fig S3A).

To rule out potential confounding effects from over-expression of SidK-mCherry, which can itself have an impact on lysosomal function (Maxson et al., 2022), we examined colocalisation of LAMP1 with ATP6V1D (Fig. 3B). In control cells, most LAMP1 colocalised with ATP6V1D as expected. This did not change after 8 hr of mycolactone exposure but, at 24 hr, both the level of membrane-associated ATP6V1D and the proportion colocalising with LAMP1 were reduced and by 32 hr there was little overlap between the signals. This was again reflected in a reduced Pearson’s R value confirming that the V_1_ complex no longer associated with lysosomes.

At 32 hr, SidK-mCherry remained associated with vesicular compartments which we hypothesised could be the Golgi. However, we also observed a loss of colocalisation between SidK-mCherry and ATP6V1D with the Golgi marker Giantin, becoming pronounced at 32 hr (Fig S3B and Fig S3C respectively). Therefore, it is not clear at the current time what these compartments are. Nevertheless, the results suggest that levels of functional V-ATPase gradually decline over time following exposure to mycolactone. The difference in kinetics between the rapid loss of ATP6AP1 and ATP6AP2 and the slower reduction in V_1_ association suggest the accessory proteins may be made in excess and it is not until they are completely lost, and pre-existing complexes are turned over, that the full impact on V-ATPase complex formation can be seen.

### 3.4 Mycolactone causes a decrease in lysosome number and acidity

**Fig. 4:**
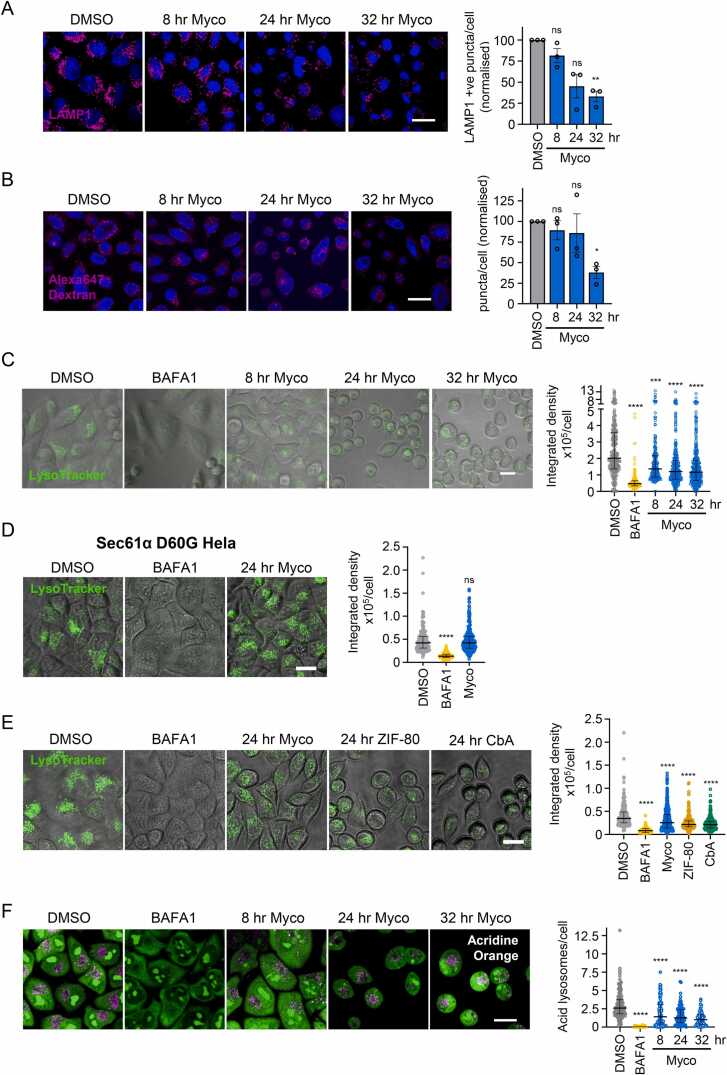
Prolonged exposure to mycolactone leads to a reduction in lysosome number and acidity. **A.** HeLa cells were incubated with DMSO or mycolactone for the indicated times, fixed with 4% PFA, permeabilised, stained with anti-LAMP1 antibodies (magenta) and counterstained with DAPI. Quantitation of mean LAMP1 positive puncta per cell. Data represents the normalised mean of 3 independent experiments ± SEM, one-sample *t*-test. **B.** HeLa cells pulsed with 0.1 mg/ml Alexa 647 dextran for 3 hr and chased for 1 hr before fixing and counterstaining with DAPI. Quantitation of mean Alexa 647 positive puncta per cell. Data represents the normalised mean of n = 3 independent experiments ± SEM (one-sample *t*-test). **C.** HeLa cells incubated as described, then incubated with 75 nM LysoTracker Green DND-26 for 10 min before washing. Cells were re-incubated 10 min then imaged. Quantitation of integrated density of LysoTracker fluorescence per cell for at least 330 cells. Data is representative of n = 3 independent experiments showing median ± IQR (Kruskal-Wallis test). **D.** HeLa cells expressing Sec61α D60G were incubated for 24 hr with DMSO or mycolactone or BAFA1 for 1 hr as described then stained with LysoTracker as above. Quantitation of integrated density of LysoTracker fluorescence per cell for at least 185 cells. Data is representative of n = 3 independent experiments showing median ± IQR (Kruskal-Wallis test). **E.** HeLa cells were incubated for 24 hr with DMSO or mycolactone or BAFA1 for 1 hr as described then stained with LysoTracker as above. Data is representative of n = 3 independent experiments showing median ± IQR (Kruskal-Wallis test). Quantitation of integrated density of LysoTracker fluorescence per cell for at least 194 cells. **F.** HeLa cells were incubated as described then incubated with 2 µM acridine orange for 30 min before washing and imaging. Acid lysosomes are pseudocoloured magenta, whereas the green is observed where the acridine orange remains in non-acidic compartments. Quantitation of acid lysosomes for at least 61 cells is shown. Data is representative of n = 3 independent experiments showing median ± IQR, Kruskal-Wallis test. All scale bars = 20 µm. ns; not significant, *; p < 0.05, **; p < 0.01, ***; p < 0.001, ****; p < 0.0001.

The effects we have observed with the LC3 tandem tag, DQ BSA and V_1_ localisation could reflect a reduction in lysosomal number, impaired acidification or both. To tease these apart, we enumerated lysosomes and found mycolactone caused a decrease in LAMP1^+ve^ vesicles in HeLa cells that became significant after 32 hr (p < 0.01; Fig. 4A). Since LAMP1 is a signal peptide-containing protein, and therefore a predicted direct target of mycolactone, we performed immunoblotting to determine whether the apparent reduction in lysosomes could be associated with loss of protein expression (Fig S4A). LAMP1 protein levels are indeed significantly reduced after mycolactone treatment. We therefore assessed lysosome number by labelling with Alexa647-dextran(Fig. 4B). Here, a significant reduction in Alexa647-dextran^+ve^ puncta (p < 0.05) was also seen at 32 hr, confirming that total lysosome numbers are indeed reduced by prolonged exposure to mycolactone.

It is possible reduced levels of LAMP1 could explain the fall in the lysosome numbers, as LAMP1 is implicated in lysosome biogenesis and can regulate pH via interaction with TMEM175 (Zhang et al., 2023). However, in contrast to our *in vitro* findings, lysosomal proteolytic activity in mice deficient in LAMP1 is unchanged (Andrejewski et al., 1999). Furthermore, while LAMP1 loss offers an explanation for the inhibition of late-stage autophagy at 32 hr, it does not explain the reduced DQ Red BSA proteolysis at earlier timepoints (8 hr, Fig. 1C). Irrespective of the mechanism, the results show that Sec61 inhibition affects lysosome biogenesis/turnover. The impact of mycolactone on lysosome numbers mirrors that seen with CbA (Shi et al., 2021) and is likely a shared effect of translocon inhibitors.

Depletion of ATP6AP1 and/or ATP6AP2 leads to defects in lysosomal acidification (Guida et al., 2018, Pareja et al., 2018, Rujano et al., 2017, Yan et al., 2025). To determine whether mycolactone has an impact on lysosomal pH we labelled cells with LysoTracker Green DND-26, which accumulates in acidic compartments. In control cells, LysoTracker staining could be seen in most cells, predominantly in the perinuclear region and the signal was profoundly depressed by BAFA1 (Fig. 4C). At 8 hr, LysoTracker could still be detected in many mycolactone-exposed cells but the median was reduced compared to the control (p < 0.001). After 24 hr, while some cells still exhibited strong fluorescence, others showed very low LysoTracker positivity. The proportion of these low stained cells increased at 32 hr but did not reach the level of inhibition seen with BAFA1. The loss of LysoTracker accumulation is a consequence of Sec61 inhibition, since no reduction in staining was seen in cells expressing Sec61α D60G but both ZIF-80 and CbA caused a significant loss of Lysotracker fluorescence after 24 hr (Fig. 4D and E).

We hypothesised the LysoTracker results were due to the effect of mycolactone on lysosomal pH. To test this, we incubated cells with acridine orange, a cell permeable dye which accumulates in lysosomes. Acridine orange emits green fluorescence in its monomeric form but in an acidic environment dimerises, causing a shift to red fluorescence emission, as seen in control conditions (Fig. 4F). This was blocked by BAFA1, as expected. On exposure to mycolactone, the number of acidic lysosomes per cell decreased over time (p < 0.001). Taken together, the results show that mycolactone reduces the number of acid lysosomes in cells and, since this precedes the reduction in total lysosomes, this may be due at least in part to impaired acidification following loss of ATP6AP1 and ATP6AP2.

*M. ulcerans* is a mycobacterium that infects macrophages as part of its lifecycle (Torrado et al., 2007). Since the endolysosomal system is critical for host defence against mycobacteria, we investigated whether mycolactone also reduced LysoTracker accumulation in BMDM and THP-1 cells (Fig S4B-C). This was indeed the case, suggesting that V-ATPase down-regulation could play a role in the infection cycle of *M. ulcerans* in Buruli ulcer disease. This is consistent with reports of reduced accumulation of LysoTracker dye in phagosomes infected with mycolactone-positive *M. ulcerans* (Torrado et al., 2010) and could represent an alternative strategy for intra-macrophage survival that explains how *M. ulcerans* can grow in host macrophages despite the absence of ESX proteins essential for *M. tuberculosis* and *M. marinum* virulence (Stinear et al., 2007, Torrado et al., 2007). Our findings therefore demonstrate a novel virulence mechanism targeting endolysosomal system acidification. Although other lysosomal proteins are downregulated by mycolactone that may have an impact individually or synergistically, the loss of ATP6AP1 and ATP6AP2 is sufficient to cause the phenotype we observe, due to their critical role in V-ATPase complex assembly.

Interestingly, ATP6AP1 has recently also been shown to be a guanine nucleotide exchange factor for Rheb, a component of mTORC1(Feng et al., 2024). Thus, its depletion may be a factor in the nuclear translocation of TFEB seen following mycolactone exposure. ATP6AP2 knockout has been shown to activate TFEB in liver (Kissing et al., 2017) but the role of ATP6AP1 in its regulation is unknown. Regardless of the underlying mechanism, the activation of lysosomal stress pathways by mycolactone is ineffective, since the cells are no longer able to respond by upregulating lysosome biogenesis and the capacity of the cell to cope with the proteostatic stress caused by Sec61 inhibition is compromised. Therefore, this virulence mechanism not only affects bacterial survival in the macrophage, but could also contribute to the cell death and tissue necrosis seen in Buruli ulcer.

The effects of Sec61 inhibition on lysosomes has broader implications, since Sec61 inhibitors are under investigation as treatments for cancer, viral infections and rheumatoid arthritis (Domenger et al., 2022, Heaton et al., 2016, Rehan et al., 2023). The long-term impact of mycolactone on lysosomal function may be a key component of its cytotoxicity that can be exploited, but could also represent a source of unwanted side effects in Sec61 targeting therapies.

## CRediT authorship contribution statement

**Hall Belinda Suzette:** Writing – review & editing, Writing – original draft, Visualization, Validation, Supervision, Methodology, Investigation, Funding acquisition, Formal analysis, Conceptualization. **Simmonds Rachel:** Writing – review & editing, Writing – original draft, Supervision, Project administration, Funding acquisition, Conceptualization. **Kwabena Owusu-Boateng:** Investigation, Formal analysis, Data curation. **Shi Wei Q:** Resources. **Kerry L McPhail:** Resources.

## Funding

This work is funded by 10.13039/501100000265MRC project grant MR/W02618X/1. KO-B is supported by a University of Surrey Vice-Chancellor’s Award.

## Declaration of Competing Interest

The authors declare that they have no known competing financial interests or personal relationships that could have appeared to influence the work reported in this paper.

## Data Availability

Raw data for all figures is available at https://data.mendeley.com/drafts/5gyvf6nf53

## Footnotes

Appendix A Supplementary data associated with this article can be found in the online version at doi:10.1016/j.ejcb.2026.151541.

## References

[bib1] Abbas Y.M., Wu D., Bueler S.A., Robinson C.V., Rubinstein J.L. (2020). Structure of V-ATPase from the mammalian brain. Science.

[bib2] Andrejewski N., Punnonen E.L., Guhde G., Tanaka Y., Lullmann-Rauch R., Hartmann D., von Figura K., Saftig P. (1999). Normal lysosomal morphology and function in LAMP-1-deficient mice. J. Biol. Chem..

[bib3] Baron L., Paatero A.O., Morel J.D., Impens F., Guenin-Macé L., Saint-Auret S., Blanchard N., Dillmann R., Niang F., Pellegrini S., Taunton J., Paavilainen V.O., Demangel C. (2016). Mycolactone subverts immunity by selectively blocking the Sec61 translocon. J. Exp. Med..

[bib4] Bhadra P., Dos Santos S., Gamayun I., Pick T., Neumann C., Ogbechi J., Hall B.S., Zimmermann R., Helms V., Simmonds R.E., Cavalié A. (2021). Mycolactone enhances the Ca2+ leak from endoplasmic reticulum by trapping Sec61 translocons in a Ca2+ permeable state. Biochem J..

[bib5] Bowman E.J., Siebers A., Altendorf K. (1988). Bafilomycins: a class of inhibitors of membrane ATPases from microorganisms, animal cells, and plant cells. Proc. Natl. Acad. Sci. USA.

[bib6] Domenger A., Choisy C., Baron L., Mayau V., Perthame E., Deriano L., Arnulf B., Bories J.C., Dadaglio G., Demangel C. (2022). The Sec61 translocon is a therapeutic vulnerability in multiple myeloma. EMBO Mol. Med.

[bib7] Feng R., Liu F., Li R., Zhou Z., Lin Z., Lin S., Deng S., Li Y., Nong B., Xia Y., Li Z., Zhong X., Yang S., Wan G., Ma W., Wu S., Songyang Z. (2024). The rapid proximity labeling system PhastID identifies ATP6AP1 as an unconventional GEF for Rheb. Cell Res.

[bib8] Gama J.B., Ohlmeier S., Martins T.G., Fraga A.G., Sampaio-Marques B., Carvalho M.A., Proenca F., Silva M.T., Pedrosa J., Ludovico P. (2014). Proteomic analysis of the action of the Mycobacterium ulcerans toxin mycolactone: targeting host cells cytoskeleton and collagen. PLoS Negl. Trop. Dis..

[bib9] George K.M., Chatterjee D., Gunawardana G., Welty D., Hayman J., Lee R., Small P.L. (1999). Mycolactone: a polyketide toxin from Mycobacterium ulcerans required for virulence. Science.

[bib10] George K.M., Pascopella L., Welty D.M., Small P.L. (2000). A Mycobacterium ulcerans toxin, mycolactone, causes apoptosis in guinea pig ulcers and tissue culture cells. Infect. Immun..

[bib11] Gerard S.F., Hall B.S., Zaki A.M., Corfield K.A., Mayerhofer P.U., Costa C., Whelligan D.K., Biggin P.C., Simmonds R.E., Higgins M.K. (2020). Structure of the inhibited state of the sec translocon. Mol. Cell.

[bib12] Guenin-Mace L., Carrette F., Asperti-Boursin F., Le Bon A., Caleechurn L., Di Bartolo V., Fontanet A., Bismuth G., Demangel C. (2011). Mycolactone impairs T cell homing by suppressing microRNA control of L-selectin expression. Proc. Natl. Acad. Sci. USA.

[bib13] Guida M.C., Hermle T., Graham L.A., Hauser V., Ryan M., Stevens T.H., Simons M. (2018). ATP6AP2 functions as a V-ATPase assembly factor in the endoplasmic reticulum. Mol. Biol. Cell.

[bib14] Hall B.S., Dos Santos S.J., Hsieh L.T., Manifava M., Ruf M.T., Pluschke G., Ktistakis N., Simmonds R.E. (2021). Inhibition of the SEC61 translocon by mycolactone induces a protective autophagic response controlled by EIF2S1-dependent translation that does not require ULK1 activity. Autophagy.

[bib15] Hall B.S., Hill K., McKenna M., Ogbechi J., High S., Willis A.E., Simmonds R.E. (2014). The pathogenic mechanism of the Mycobacterium ulcerans virulence factor, mycolactone, depends on blockade of protein translocation into the ER. PLoS Pathog..

[bib16] Hau A.M., Greenwood J.A., Lohr C.V., Serrill J.D., Proteau P.J., Ganley I.G., McPhail K.L., Ishmael J.E. (2013). Coibamide A induces mTOR-independent autophagy and cell death in human glioblastoma cells. PLoS One.

[bib17] Heaton N.S., Moshkina N., Fenouil R., Gardner T.J., Aguirre S., Shah P.S., Zhao N., Manganaro L., Hultquist J.F., Noel J., Sachs D., Hamilton J., Leon P.E., Chawdury A., Tripathi S., Melegari C., Campisi L., Hai R., Metreveli G., Gamarnik A.V., Garcia-Sastre A., Greenbaum B., Simon V., Fernandez-Sesma A., Krogan N.J., Mulder L.C.F., van Bakel H., Tortorella D., Taunton J., Palese P., Marazzi I. (2016). Targeting viral proteostasis limits Influenza Virus, HIV, and Dengue Virus infection. Immunity.

[bib18] Hsieh L.T., Hall B.S., Newcombe J., Mendum T.A., Varela S.S., Umrania Y., Deery M.J., Shi W.Q., Diaz-Delgado J., Salguero F.J., Simmonds R.E. (2025). The Mycobacterium ulcerans toxin mycolactone causes destructive Sec61-dependent loss of the endothelial glycocalyx and vessel basement membrane to drive skin necrosis. Elife.

[bib19] Itskanov S., Wang L., Junne T., Sherriff R., Xiao L., Blanchard N., Shi W.Q., Forsyth C., Hoepfner D., Spiess M., Park E. (2023). A common mechanism of Sec61 translocon inhibition by small molecules. Nat. Chem. Biol..

[bib20] Jansen E.J., Timal S., Ryan M., Ashikov A., van Scherpenzeel M., Graham L.A., Mandel H., Hoischen A., Iancu T.C., Raymond K., Steenbergen G., Gilissen C., Huijben K., van Bakel N.H., Maeda Y., Rodenburg R.J., Adamowicz M., Crushell E., Koenen H., Adams D., Vodopiutz J., Greber-Platzer S., Muller T., Dueckers G., Morava E., Sykut-Cegielska J., Martens G.J., Wevers R.A., Niehues T., Huynen M.A., Veltman J.A., Stevens T.H., Lefeber D.J. (2016). ATP6AP1 deficiency causes an immunodeficiency with hepatopathy, cognitive impairment and abnormal protein glycosylation. Nat. Commun..

[bib21] Kissing S., Rudnik S., Damme M., Lullmann-Rauch R., Ichihara A., Kornak U., Eskelinen E.L., Jabs S., Heeren J., De Brabander J.K., Haas A., Saftig P. (2017). Disruption of the vacuolar-type H(+)-ATPase complex in liver causes MTORC1-independent accumulation of autophagic vacuoles and lysosomes. Autophagy.

[bib22] Klionsky D.J., Abdel-Aziz A.K., Abdelfatah S., Abdellatif M., Abdoli A., Abel S., Abeliovich H., Abildgaard M.H., Abudu Y.P., Acevedo-Arozena A., Adamopoulos I.E., Adeli K., Adolph T.E., Adornetto A., Aflaki E., Agam G., Agarwal A., Aggarwal B.B., Agnello M., Agostinis P., N J., Agrewala, Agrotis A., Aguilar P.V., Ahmad S.T., Ahmed Z.M., Ahumada-Castro U., Aits S., Aizawa S., Akkoc Y., Akoumianaki T., Akpinar H.A., Al-Abd A.M., Al-Akra L., Al-Gharaibeh A., Alaoui-Jamali M.A., Alberti S., Alcocer-Gómez E., Alessandri C., Ali M., Alim Al-Bari M.A., Aliwaini S., Alizadeh J., Almacellas E., Almasan A., Alonso A., Alonso G.D., Altan-Bonnet N., Altieri D.C., Álvarez É M.C., Alves S., Alves da Costa C., Alzaharna M.M., Amadio M., Amantini C., Amaral C., Ambrosio S., Amer A.O., Ammanathan V., An Z., Andersen S.U., Andrabi S.A., Andrade-Silva M., Andres A.M., Angelini S., Ann D., Anozie U.C., Ansari M.Y., Antas P., Antebi A., Antón Z., Anwar T., Apetoh L., Apostolova N., Araki T., Araki Y., Arasaki K., Araújo W.L., Araya J., Arden C., Arévalo M.A., Arguelles S., Arias E., Arikkath J., Arimoto H., Ariosa A.R., Armstrong-James D., Arnauné-Pelloquin L., Aroca A., Arroyo D.S., Arsov I., Artero R., Asaro D.M.L., Aschner M., Ashrafizadeh M., Ashur-Fabian O., Atanasov A.G., Au A.K., Auberger P., Auner H.W., Aurelian L. (2021). Guidelines for the use and interpretation of assays for monitoring autophagy (4th edition). Autophagy.

[bib23] Marion E., Song O.R., Christophe T., Babonneau J., Fenistein D., Eyer J., Letournel F., Henrion D., Clere N., Paille V., Guerineau N.C., Saint Andre J.P., Gersbach P., Altmann K.H., Stinear T.P., Comoglio Y., Sandoz G., Preisser L., Delneste Y., Yeramian E., Marsollier L., Brodin P. (2014). Mycobacterial toxin induces analgesia in buruli ulcer by targeting the angiotensin pathways. Cell.

[bib24] Maxson M.E., Abbas Y.M., Wu J.Z., Plumb J.D., Grinstein S., Rubinstein J.L. (2022). Detection and quantification of the vacuolar H+ATPase using the Legionella effector protein SidK. J. Cell Biol..

[bib25] McKenna M., Simmonds R.E., High S. (2017). Mycolactone reveals the substrate-driven complexity of Sec61-dependent transmembrane protein biogenesis. J. Cell Sci..

[bib26] Nelson N., Harvey W.R. (1999). Vacuolar and plasma membrane proton-adenosinetriphosphatases. Physiol. Rev..

[bib27] Ogbechi J., Hall B.S., Sbarrato T., Taunton J., Willis A.E., Wek R.C., Simmonds R.E. (2018). Inhibition of Sec61-dependent translocation by mycolactone uncouples the integrated stress response from ER stress, driving cytotoxicity via translational activation of ATF4. Cell Death Dis..

[bib28] Ogbechi J., Ruf M.T., Hall B.S., Bodman-Smith K., Vogel M., Wu H.L., Stainer A., Esmon C.T., Ahnstrom J., Pluschke G., Simmonds R.E. (2015). Mycolactone-dependent depletion of endothelial cell thrombomodulin is strongly associated with fibrin deposition in Buruli Ulcer Lesions. PLoS Pathog..

[bib29] Pankiv S., Clausen T.H., Lamark T., Brech A., Bruun J.A., Outzen H., Overvatn A., Bjorkoy G., Johansen T. (2007). p62/SQSTM1 binds directly to Atg8/LC3 to facilitate degradation of ubiquitinated protein aggregates by autophagy. J. Biol. Chem..

[bib30] Pareja F., Brandes A.H., Basili T., Selenica P., Geyer F.C., Fan D., Da Cruz Paula A., Kumar R., Brown D.N., Gularte-Merida R., Alemar B., Bi R., Lim R.S., de Bruijn I., Fujisawa S., Gardner R., Feng E., Li A., da Silva E.M., Lozada J.R., Blecua P., Cohen-Gould L., Jungbluth A.A., Rakha E.A., Ellis I.O., Edelweiss M.I.A., Palazzo J., Norton L., Hollmann T., Edelweiss M., Rubin B.P., Weigelt B., Reis-Filho J.S. (2018). Loss-of-function mutations in ATP6AP1 and ATP6AP2 in granular cell tumors. Nat. Commun..

[bib31] Pena-Llopis S., Vega-Rubin-de-Celis S., Schwartz J.C., Wolff N.C., Tran T.A., Zou L., Xie X.J., Corey D.R., Brugarolas J. (2011). Regulation of TFEB and V-ATPases by mTORC1. EMBO J..

[bib32] Qi C., Lei L., Hu J., Wang G., Liu J., Ou S. (2022). Identification of a five-gene signature deriving from the vacuolar ATPase (V-ATPase) sub-classifies gliomas and decides prognoses and immune microenvironment alterations. Cell Cycle.

[bib33] Raben N., Puertollano R. (2016). TFEB and TFE3: linking lysosomes to cellular adaptation to stress. Annu Rev. Cell Dev. Biol..

[bib34] Rehan S., Tranter D., Sharp P.P., Craven G.B., Lowe E., Anderl J.L., Muchamuel T., Abrishami V., Kuivanen S., Wenzell N.A., Jennings A., Kalyanaraman C., Strandin T., Javanainen M., Vapalahti O., Jacobson M.P., McMinn D., Kirk C.J., Huiskonen J.T., Taunton J., Paavilainen V.O. (2023). Signal peptide mimicry primes Sec61 for client-selective inhibition. Nat. Chem. Biol..

[bib35] Rujano M.A., Cannata Serio M., Panasyuk G., Peanne R., Reunert J., Rymen D., Hauser V., Park J.H., Freisinger P., Souche E., Guida M.C., Maier E.M., Wada Y., Jager S., Krogan N.J., Kretz O., Nobre S., Garcia P., Quelhas D., Bird T.D., Raskind W.H., Schwake M., Duvet S., Foulquier F., Matthijs G., Marquardt T., Simons M. (2017). Mutations in the X-linked ATP6AP2 cause a glycosylation disorder with autophagic defects. J. Exp. Med.

[bib36] Sardiello M., Palmieri M., di Ronza A., Medina D.L., Valenza M., Gennarino V.A., Di Malta C., Donaudy F., Embrione V., Polishchuk R.S., Banfi S., Parenti G., Cattaneo E., Ballabio A. (2009). A gene network regulating lysosomal biogenesis and function. Science.

[bib37] Shi W., Lu D., Wu C., Li M., Ding Z., Li Y., Chen B., Lin X., Su W., Shao X., Xia Z., Fang L., Liu K., Li H. (2021). Coibamide A kills cancer cells through inhibiting autophagy. Biochem Biophys. Res Commun..

[bib38] Stinear T.P., Seemann T., Pidot S., Frigui W., Reysset G., Garnier T., Meurice G., Simon D., Bouchier C., Ma L., Tichit M., Porter J.L., Ryan J., Johnson P.D., Davies J.K., Jenkin G.A., Small P.L., Jones L.M., Tekaia F., Laval F., Daffe M., Parkhill J., Cole S.T. (2007). Reductive evolution and niche adaptation inferred from the genome of Mycobacterium ulcerans, the causative agent of Buruli ulcer. Genome Res..

[bib39] Torrado E., Fraga A.G., Castro A.G., Stragier P., Meyers W.M., Portaels F., Silva M.T., Pedrosa J. (2007). Evidence for an intramacrophage growth phase of Mycobacterium ulcerans. Infect. Immun..

[bib40] Torrado E., Fraga A.G., Logarinho E., Martins T.G., Carmona J.A., Gama J.B., Carvalho M.A., Proenca F., Castro A.G., Pedrosa J. (2010). IFN-gamma-dependent activation of macrophages during experimental infections by Mycobacterium ulcerans is impaired by the toxin mycolactone. J. Immunol..

[bib41] Tranter D., Paatero A.O., Kawaguchi S., Kazemi S., Serrill J.D., Kellosalo J., Vogel W.K., Richter U., Mattos D.R., Wan X., Thornburg C.C., Oishi S., McPhail K.L., Ishmael J.E., Paavilainen V.O. (2020). Coibamide A targets Sec61 to prevent biogenesis of secretory and membrane proteins. ACS Chem. Biol..

[bib42] Wang L., Wu D., Robinson C.V., Wu H., Fu T.M. (2020). Structures of a complete human V-ATPase reveal mechanisms of its assembly. Mol. Cell.

[bib43] Xu L., Shen X., Bryan A., Banga S., Swanson M.S., Luo Z.Q. (2010). Inhibition of host vacuolar H+-ATPase activity by a Legionella pneumophila effector. PLoS Pathog..

[bib44] Yan Z., Huang A., Ma D., Hong C., Zhang S., He L., Rao H., Luo S. (2025). ATP6AP1 promotes cell proliferation and tamoxifen resistance in luminal breast cancer by inducing autophagy. Cell Death Dis..

[bib45] Yotsu R.R., Suzuki K., Simmonds R.E., Bedimo R., Ablordey A., Yeboah-Manu D., Phillips R., Asiedu K. (2018). Buruli Ulcer: a review of the current knowledge. Curr. Trop. Med. Rep..

[bib46] Zhang J., Zeng W., Han Y., Lee W.R., Liou J., Jiang Y. (2023). Lysosomal LAMP proteins regulate lysosomal pH by direct inhibition of the TMEM175 channel. Mol. Cell.

[bib47] Zong G., Hu Z., Duah K.B., Andrews L.E., Zhou J., O'Keefe S., Whisenhunt L., Shim J.S., Du Y., High S., Shi W.Q. (2020). Ring expansion leads to a more potent analogue of ipomoeassin F. J. Org. Chem..

