# Supplementary material for: A mycobacterial Sec61 inhibitor disrupts lysosome function by blocking Vacuolar-ATPase biosynthesis
Source: Eur J Cell Biol. 2026 Sep;105(3):151541. doi: 10.1016/j.ejcb.2026.151541 (PMC13468756; doi:10.1016/j.ejcb.2026.151541)
Supplement: Supplementary file 1 [file mmc1.docx]

**Supplementary Figure Legends**

**Figure S1 Mycolactone blocks late-stage autophagy, disrupts lysosomal function and activates TFEB signalling pathway.**

**A**. Schematic of LC3 tandem tag assay. **B.** Cells were transiently transfected with pDEST-mcherry-eGFP-LC3B before treatment with mycolactone as described in Fig1A. Quantitation shows the total number of magenta and white puncta per cell. Data represents the mean of 3 independent experiments ± SEM analysed by RM 2-way ANOVA. Data are compared to DMSO in the absence and presence of BAFA1 separately, and the effect of BAFA1 on each treatment is compared (in blue). **C.** HeLa cells were incubated with 0.05% DMSO or 31.25ng/ml mycolactone (Myco) for various times, stained with CellEvent and propidium iodide and imaged. Data represents the % viable cells at each time point and is the mean of n = 3 independent experiments ± SEM (RM one-way ANOVA). **D.** BMDM were seeded onto IBIDI 8-well chamber-slides. THP-1 cells were plated onto IBIDI 8-well chamber slides in the presence of 20ng/ml PMA. After 72 hr medium was replaced and cells incubated for 24 hr Cells were incubated with 0.05% DMSO for 24 hr, 31.25ng/ml mycolactone (Myco) for various times or 200nM Bafilomycin A1 (BAFA1) for 4 hr. DS Red BSA was added for 4hr before washing and imaging. Scale bar = 50µm. **E.** Quantitation of median DS Red BSA fluorescence per cell for at least 43 cells (BMDM) or 53 cells (THP-1) ± IQR. Data representative of n = 3 independent experiments (Kruskal-Wallis test. **F.** WT or Sec61α D60G expressing Hela cells were incubated for 20hr with DMSO, Myco or BAFA1 then with 20µg/ml DQ Red BSA for 4hr before washing and imaging. Images are representative of 3 independent experiments. Scale bar = 20µm. **G.** Hela cells were incubated for 20hr with DMSO, Myco, Zif80, CbA or BAFA1 then with 20µg/ml DQ Red BSA for 4 hr before washing and imaging. Scale bar = 20µm. **H.** WT or Sec61α D60G expressing Hela cells were incubated with DMSO or mycolactone for various times, then fixed, permeabilised and stained with anti-TFEB antibodies (white). Slides were counterstained with DAPI. Scale bar = 10μm. **I.** Quantitation of mean % cells with nuclear TFEB staining. Three fields containing at least 50 cells were counted for each treatment. Data represents the mean values from n = 3 biological replicates ± SEM (2-way ANOVA). **J.** Hela cells were incubated with DMSO or mycolactone, Zif80 or CbA for 24hr, then fixed, permeabilised and stained with anti-TFEB antibodies (white). Slides were counterstained with DAPI. Scale bar = 10μm. **K.** Quantitation of mean % cells with nuclear TFEB staining. Three fields containing at least 57 cells were counted for each treatment. Data represents the mean values from n=3 biological replicates ± SEM (1-way ANOVA). ). ns; not significant, *; p<0.05, **; p<0.01, ***; p<0.001, ****; p<0.0001 or as indicated.

**Figure S2 Mycolactone targets the ATP6A1 and ATP6A2 subunits of the Vacuolar ATPase.**

**A.** Immunoblot of HeLa cell lysates incubated with 0.05% DMSO or mycolactone for various times and stained with anti-Rab7 antibody, with anti-GAPDH used as a loading control. Data is representative of n = 2 biological repeats. **B.** Immunoblot of THP-1 cell lysates incubated with 0.05% DMSO or mycolactone for various times and stained with anti-ATP6AP1 and -ATP6AP2 with anti-GAPDH used as a loading control. Graph shows signal intensity levels relative to GAPDH. Values represent the means of n=3 independent experiments ±SEM for ATP6AP1 (1-way ANOVA) and 2 independent experiments ± range for ATP6AP2 **C.** Quantitation of expression relative to GAPDH for blots shown in Fig 2D. Values represent the means of n=3 independent experiments ± SEM (2-way ANOVA). **D.** Quantitation of expression relative to GAPDH for blots shown in Fig 2D. Values represent the means of n=3 independent experiments ± SEM (1-sample t test). **, p<0.01; ***, p<0.001; ****, p<0.0001.

**Figure S3 Reduced association of V-ATPase V_1_ with lysosomes.**

**A.** HeLa cells transiently transfected with pYMA1_SidK_mCH were incubated with 0.05% DMSO for 32hr or 31.25ng/ml mycolactone (Myco) for various times. Cells were pulsed with 0.1mg/ml Alexa 647 dextran (magenta) for 3hr then chased for 1hr before fixing. mCherry Sidk is pseudocoloured in green. Data is representative of n=2 independent experiments. **B.** HeLa cells transiently transfected with pYMA1_SidK_mCH were incubated with 0.05% DMSO for 32hr or 31.25ng/ml mycolactone (Myco) for various times before fixing and staining with anti-human Giantin antibodies (green) and counterstaining with DAPI with mCherry Sidk pseudocoloured in magenta. Scale bar = 20µm. Images are representative of n = 3 independent experiments. **C.** HeLa cells were incubated with 0.05% DMSO for 32hr or 31.25ng/ml mycolactone (Myco) for various times, fixed with methanol, blocked and stained with anti-ATP6V1D (green) and anti-Giantin antibodies (magenta) then counterstained with DAPI. Scale bar = 20µm. Images are representative of n = 2 independent experiments.

**Figure S4 Prolonged exposure to mycolactone leads to a reduction in lysosome number and acidity**

**A.** Immunoblot of HeLa cell lysates incubated with 0.05% DMSO or mycolactone for various times and stained with anti-LAMP1 antibody, with anti-GAPDH used as a loading control. Graph represent the mean band intensity relative to GAPDH from 3 biological repeats ± SEM (1-sample t test). **B.** BMDM were seeded onto IBIDI 8-well chamberslides and incubated with 0.05% DMSO for 24hr, 31.25ng/ml mycolactone (Myco) for various times or 200nM Bafilomycin A1 (BAFA1) for 4 hr, then incubated with 75nM Lysotracker Green DND-26 for 10min before washing. Cells were re-incubated 10min then imaged. Scale bar = 50µm. Data represents median of integrated density of Lysotracker fluorescence per cell for at least 74 cells ± IQR. Data representative of n = 2 independent experiments. **C.** THP-1 cells were plated onto IBIDI 8-well chamber slides in the presence of 20ng/ml PMA. After 72hr medium was replaced and cells reincubated for 24hr before adding 0.05% DMSO for 24hr, 31.25ng/ml mycolactone (Myco) for various times or 200nM Bafilomycin A1 (BAFA1) for 4hr, then incubated with 75nM Lysotracker Green DND-26 for 10min before washing. Cells were re-incubated 10min then imaged. Scale bar = 50µm. Data represents median of integrated density of Lysotracker fluorescence per cell for at least 41 cells ± IQR. Data is representative of n=3 independent experiments. All statistical analysis was carried out using Kruskal-Wallis test. ns; not significant, *; p<0.05, **; p<0.01, ***; p<0.001, ****; p<0.0001.
